# Supplementary figures and images for: Unexpected differences in the pharmacokinetics of N-acetyl-DL-leucine enantiomers after oral dosing and their clinical relevance
Source: PLoS One. 2020 Feb 27;15(2):e0229585. doi: 10.1371/journal.pone.0229585 (PMC7046201; doi:10.1371/journal.pone.0229585)

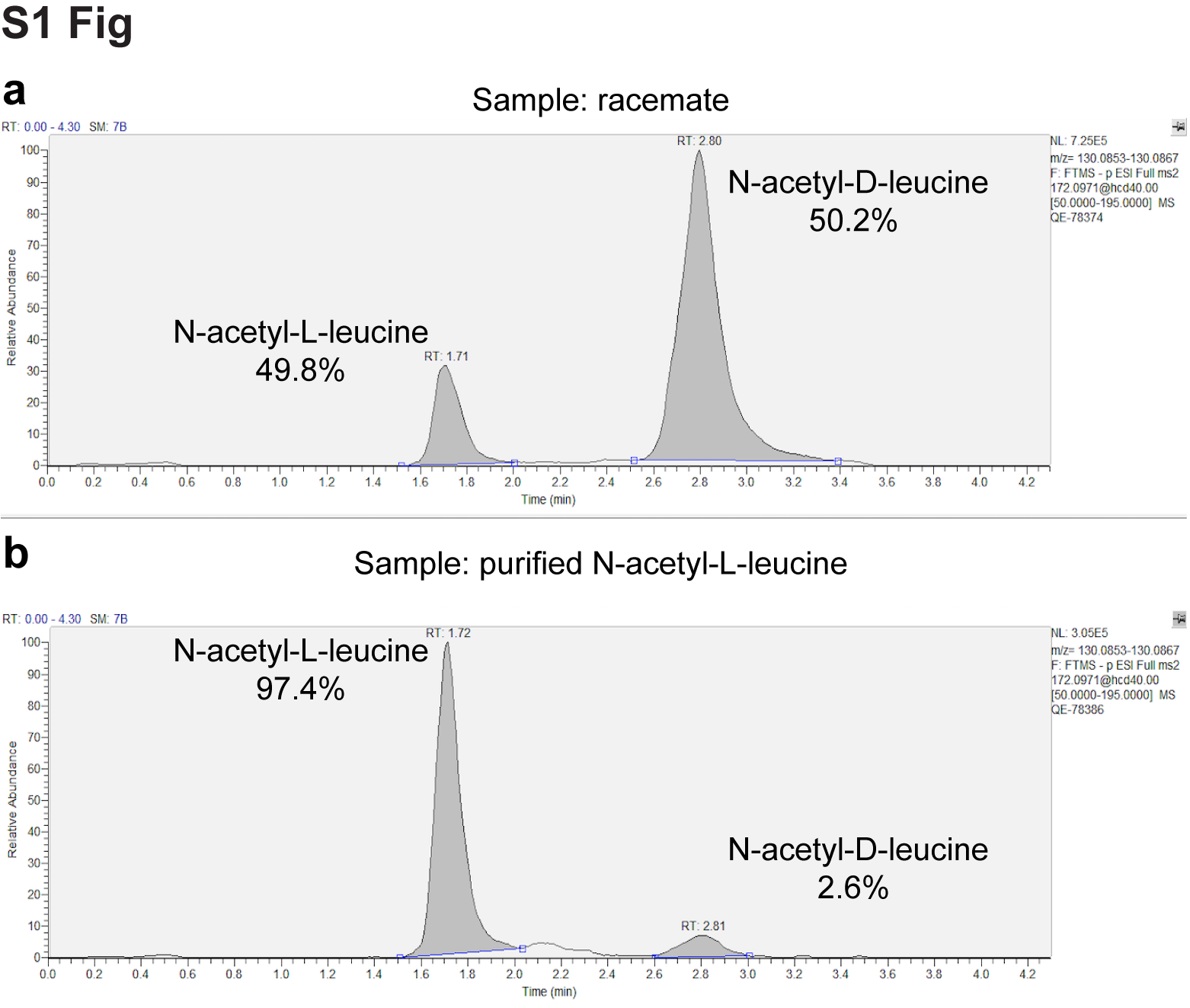

Supplement: S1 Fig — (a) Spectrum of racemate. (b) Spectrum of purified N-acetyl-L-leucine. Note that the peak areas are not directly comparable with concentration due to differences in the extent of ionization of the compound in the mass spectrometers ionization chamber due to relative differences in aqueous and organic solvent concentrations in the mobile phase at those time points due to a gradient elution. Therefore, quantification was based on a standard curve specific to each enantiomer. The result is that the racemate contained 50.2% N-acetyl-D-leucine and 49.8% N-acetyl-L-leucine. The purified N-acetyl-L-leucine contained 2.6% N-acetyl-D-leucine and 97.4% N-acetyl-L-leucine. The limit of detection and the limit of quantification was, respectively, 10 ng/mL and 25 ng/mL for N-acetyl-D-leucine, and 25 ng/mL and 50 ng/mL for N-acetyl-L-leucine. (TIF) [file pone.0229585.s001.tif]
