# Supplementary material for: Unexpected differences in the pharmacokinetics of N-acetyl-DL-leucine enantiomers after oral dosing and their clinical relevance
Source: PLoS One. 2020 Feb 27;15(2):e0229585. doi: 10.1371/journal.pone.0229585 (PMC7046201; doi:10.1371/journal.pone.0229585)
Supplement: S1 Table — (DOCX) [file pone.0229585.s002.docx]

**S1 Table. Measured concentrations of N-Acetyl-L-Leucine and N-Acetyl-D-Leucine in mouse plasma and tissues after p.o administration N-Acetyl-DL-Leucine at 100 mg/kg.**

| **Mouse ID** | **Sample Time**  **(h)** | **N-Acetyl-L-Leucine (ng/ml)** | | | **N-Acetyl-D-Leucine (ng/ml)** | | |
| --- | --- | --- | --- | --- | --- | --- | --- |
|  |  | **plasma** | **brain** | **muscle** | **plasma** | **brain** | **muscle** |
| 1 | 0.15 | 3030 |  |  | 93900 |  |  |
| 2 | 0.15 | 4750 |  |  | 103000 |  |  |
| 3 | 0.15 | 2460 |  |  | 61300 |  |  |
| 1 | 0.30 | 1840 | ND | 469 | 38300 | 357 | 2860 |
| 2 | 0.30 | 2730 | ND | 605 | 76200 | 491 | 4370 |
| 3 | 0.30 | 1490 | ND | 566 | 41800 | 888 | 3590 |
| 4 | 1.00 | 465 |  |  | 11500 |  |  |
| 5 | 1.00 | 1410 |  |  | 13500 |  |  |
| 6 | 1.00 | 832 |  |  | 23000 |  |  |
| 4 | 2.00 | 562 | ND | 364 | 1050 | <LoQ | 335 |
| 5 | 2.00 | <LoQ | ND | 154 | 939 | <LoQ | 396 |
| 6 | 2.00 | 683 | ND | 193 | 4320 | 117 | 829 |
| 7 | 4.00 | <LoQ |  |  | 362 |  |  |
| 8 | 4.00 | <LoQ |  |  | 329 |  |  |
| 9 | 4.00 | <LoQ |  |  | 287 |  |  |
| 7 | 6.00 | <LoQ | ND | 108 | 357 | ND | ND |
| 8 | 6.00 | <LoQ | ND | 159 | 210 | ND | ND |
| 9 | 6.00 | <LoQ | ND | 189 | 529 | ND | ND |
| 10 | 8.00 | <LoQ |  |  | 200 |  |  |
| 11 | 8.00 | <LoQ |  |  | 380 |  |  |
| 12 | 8.00 | <LoQ |  |  | 161 |  |  |
| 10 | 24.0 | <LoQ | ND | 136 | <LoQ | ND | ND |
| 11 | 24.0 | <LoQ | ND | 195 | <LoQ | ND | ND |
| 12 | 24.0 | <LoQ | ND | 153 | <LoQ | ND | ND |
| 13 | 32.0 | <LoQ |  |  | <LoQ |  |  |
| 14 | 32.0 | <LoQ |  |  | <LoQ |  |  |
| 15 | 32.0 | <LoQ |  |  | <LoQ |  |  |
| 13 | 48.0 | <LoQ | ND | 124 | <LoQ | ND | ND |
| 14 | 48.0 | <LoQ | ND | 102 | <LoQ | ND | ND |
| 15 | 48.0 | <LoQ | ND | ND | <LoQ | ND | ND |

<LoQ – value below the limit of quantification; ND – not detected
