# Supplementary material for: Unexpected differences in the pharmacokinetics of N-acetyl-DL-leucine enantiomers after oral dosing and their clinical relevance
Source: PLoS One. 2020 Feb 27;15(2):e0229585. doi: 10.1371/journal.pone.0229585 (PMC7046201; doi:10.1371/journal.pone.0229585)
Supplement: S2 Table — (DOCX) [file pone.0229585.s003.docx]

**S2 Table. Measured concentrations of N-Acetyl-L-Leucine and N-Acetyl-D-Leucine in mouse plasma and tissues after p.o administration N-Acetyl-L-Leucine at 100 mg/kg.**

| **Mouse ID** | **Sample Time**  **(h)** | **N-Acetyl-L-Leucine (ng/ml)** | | | **N-Acetyl-D-Leucine (ng/ml)** | | |
| --- | --- | --- | --- | --- | --- | --- | --- |
|  |  | **plasma** | **brain** | **muscle** | **plasma** | **brain** | **muscle** |
| 16 | 0.15 | 9090 |  |  | 582 |  |  |
| 17 | 0.15 | 12400 |  |  | 391 |  |  |
| 18 | 0.15 | 28900 |  |  | 336 |  |  |
| 16 | 0.30 | 7510 | ND | ND | 392 | ND | 1090 |
| 17 | 0.30 | 7520 | ND | ND | 463 | ND | 1110 |
| 18 | 0.30 | 10500 | ND | ND | 388 | ND | 1520 |
| 19 | 1.00 | 1590 |  |  | 116 |  |  |
| 20 | 1.00 | 4280 |  |  | 144 |  |  |
| 21 | 1.00 | 5300 |  |  | 248 |  |  |
| 19 | 2.00 | 73 | ND | ND | <LoQ | ND | 112 |
| 20 | 2.00 | 663 | ND | ND | 33.4 | ND | 119 |
| 21 | 2.00 | 502 | ND | ND | 45.6 | ND | 109 |
| 22 | 4.00 | 131 |  |  | <LoQ |  |  |
| 23 | 4.00 | 139 |  |  | <LoQ |  |  |
| 24 | 4.00 | 304 |  |  | <LoQ |  |  |
| 22 | 6.00 | 222 | ND | ND | 23.3 | ND | ND |
| 23 | 6.00 | <LoQ | ND | ND | <LoQ | ND | ND |
| 24 | 6.00 | 114 | ND | ND | 21.4 | ND | ND |
| 25 | 8.00 | <LoQ |  |  | 17.3 |  |  |
| 26 | 8.00 | <LoQ |  |  | 15.0 |  |  |
| 27 | 8.00 | <LoQ |  |  | <LoQ |  |  |
| 25 | 24.0 | <LoQ | ND | ND | <LoQ | ND | ND |
| 26 | 24.0 | <LoQ | ND | ND | <LoQ | ND | ND |
| 27 | 24.0 | <LoQ | ND | ND | <LoQ | ND | ND |
| 28 | 32.0 | <LoQ |  |  | <LoQ |  |  |
| 29 | 32.0 | <LoQ |  |  | <LoQ |  |  |
| 30 | 32.0 | <LoQ |  |  | <LoQ |  |  |
| 28 | 48.0 | <LoQ | ND | ND | <LoQ | ND | ND |
| 29 | 48.0 | <LoQ | ND | ND | <LoQ | ND | ND |
| 30 | 48.0 | <LoQ | ND | ND | <LoQ | ND | ND |

<LoQ – value below the limit of quantification
